# Structural and Micromechanical Properties of Nd:YAG Laser Marking Stainless Steel (AISI 304 and AISI 316)

**DOI:** 10.3390/ma13092168

**Published:** 2020-05-08

**Authors:** Piotr Dywel, Robert Szczesny, Piotr Domanowski, Lukasz Skowronski

**Affiliations:** 1 Faculty of Mechanical Engineering, UTP University of Science and Technology, Kaliskiego 7, 85-796 Bydgoszcz, Poland; piotr.domanowski@utp.edu.pl; 2 Faculty of Chemistry, Nicolaus Copernicus University in Torun, Gagarina 7, 87-100 Torun, Poland; robert.szczesny@umk.pl; 3 Institute of Mathematics and Physics, UTP University of Science and Technology, Kaliskiego 7, 85-796 Bydgoszcz, Poland; lukasz.skowronski@utp.edu.pl

**Keywords:** laser marking, stainless steel, micro-mechanical properties, nanoindentation testing

## Abstract

The purpose of this study is to examine the microstructure and micromechanical properties of pulsed-laser irradiated stainless steel. The laser marking was conducted for AISI 304 and AISI 316 stainless steel samples through a Nd:YAG (1064 nm) laser. The influence of process parameters such as the pulse repetition rate and scanning speed have been considered. The microstructures of obtained samples were analyzed using confocal optical microscopy (COM). The continuous stiffness measurements (CSM) technique was applied for nanoindentional hardness and elastic modulus determination. The phase compositions of obtained specimens were characterized by X-ray diffraction (XRD) and confirmed by Raman spectroscopy. The results revealed that surface roughness is directly related to overlapping distance and the energy provided by a single pulse. The hardness of irradiated samples changes significantly with the indentation depth. The instrumental hardness H_IT_ and elastic modulus E_IT_ drop sharply with the rise of the indentation depth. Thus, the hardness enhancement can be observed as the indentation depth varies between 100–1000 nm for all exanimated samples. The maximum values of H_IT_ and E_IT_ were evaluated for the region of small depths (100–200 nm). The XRD results reveal the presence of iron and chromium oxides due to irradiation, which indicates a surface hardening effect.

## 1. Introduction

Recently, laser surface treatment has become one of the most popular techniques to enhance metallic materials’ surface functional properties, such as optical properties (color marking) and corrosion resistance, as well as micromechanical properties like wear and microhardness. Among several laser-induced microstructure modification techniques, more recent attention has focused on the laser surface melting (LSM) technique, inducing deep changes in the microstructure of a treated substrate [1,2,3,4,5,6]. Although the LSM processing and other continuous laser irradiation techniques find a lot of applications [7,8,9], the pulsed laser modification technique becomes very promising. Thus, an increasing number of authors [10,11,12,13] have reported improvement of the surface properties by means of short pulsed laser beams’ interaction with the material surface. The modulation of laser pulse duration from conventional micro and nanosecond to ultrafast femtosecond and the accompanying changes in the thermal effects can lead to surface oxidation and the formation of non-equilibrium compounds [14]. Preliminary works [15] on nanocrystaline metallic coatings revealed the improvement in mechanical properties of surface-formed nanomorphologies compared to untreated coarse-grained equivalents. Once the direct correlation between penetration depth and laser irradiation energy can be observed, the femtosecond laser pretends to be more efficient in terms of hardness improvements than conventional ones [16]. The femtosecond lasers generate a shock wave pressure of up to 1000 GPa, and operate with pulse energy in the mJ range, whereas the nanosecond delivers wave pressure from just 1 to 10 GPa, and the pulse energy in the J range [17]. In general, it has commonly been assumed that surface treatment such as laser engraving or laser marking increase the lifetime and durability of proceeded components by changing their surface morphology, chemical composition and microstructure [12]. It is achieved by means of short pulsed laser beams’ interaction with the material surface, and results in rapid phase and microstructure modification, mainly due to fast heating, melting, evaporating and solidification [12]. Depending on laser marking process parameters, various oxide layer formation and characteristic cracks resulting from high temperature stress relief can be observed [18]. 

Among others, various grade stainless steel such as AISI 304 and AISI 316 are frequently influenced by the laser treatment aimed at improving robust level of protection against corrosion, as well as improving its hardness and wear. It derives directly from the ubiquity of stainless steel application and outstanding mechanical properties. Stainless steel has found many applications in the field of modern architecture, as well as the automotive, transportation, medicine and even food industries [19,20,21].

Valette et al. [22] reported that 316 stainless steel irradiated through Ti:Sa femto-second laser demonstrates an improvement of the pitting corrosion resistance. Moreover, many studies were devoted to investigate the effect of laser beams on corrosion resistance enhancement [1,3,4,23,24,25]. Laser coloring metal techniques directly affect the optical properties of an irradiated surface. There have been a number of longitude studies describing the influence of laser marking strategies on optical properties, such as obtaining color and sensitivity [10,26,27,28]. One recent study by Antonczak et al. [10] examined the correlation between the stainless steel laser marking strategy, and obtained color in terms of its repeatability. 

Considering the surface laser-microhardening effect, one must take into account the interaction of laser beams with metals resulting in laser-induced periodic surface structure formations, called LIPPS, or ripples [29,30]. These typical microstructures are nothing but surface irregularities induced by different grain-like morphology formations. Moreover, the mechanism of laser surface hardening is closely related to a rapid increase of the surface temperature, exceeding the steel austinizing threshold, and the quick cooling down caused by direct contact with a cold substrate. Several studies on the femtosecond laser-microhardening effect revealed that in most cases, hardness improvement does not exceed an increase of 20% [16]. Although extensive research has been carried out on laser marking surface modification, only few of them focus on microhardness evaluation. Cui et al. [31] discuss the influence of hexagonal oxide formation on AISI 304 treated by means of a Nd:YAG pulsed laser surface melting on surface microhardness.

The aim of this study is to investigate the microstructure and micromechanical properties of commonly used stainless steel, i.e., AISI 304 and 316, after Nd:YAG (1064 nm) pulsed laser surface modification. The paper covers in detail how the process parameters affects the phase composition and different morphology formation, resulting in hardness and elastic modulus enhancement. 

## 2. Materials and Methods 

### Experimental

For the purpose of this study, the AISI 304 and 316 stainless steel plates (1 mm-thick) have been cut into square samples of dimensions 16 mm × 16 mm. The chemical compositions of AISI 304 and 316 are given in Table 1.

Tested stainless steel plates were laser proceeded in air through a Nd:YAG pulsed laser (wavelength 1064 nm). For the laser marking purpose, the TruMark 3020 marking station (TRUMPF, Grüsch, Switzerland), with a laser source pulse duration of 20 ns, was involved. The M^2^ laser beam quality factor does not exceed 1.5. The dimension of the irradiated area was defined by a square of 12 mm × 12 mm. The pulse repetition rate, as well as the scanning speed, were varied from 10 to 1000 kHz and from 20 mm/s to 80 mm/s, respectively. Each sample was mounted on an adjustable X-Y axis table, which allows the recording of the sample displacement with an accuracy of 0.01 mm. The process parameters of obtained samples are summarized in Table 2 (the selection of samples was driven by the most noticeable changes that occur in proceeded material under various laser process parameter conditions).

The X-ray diffraction (XRD) patterns of stainless steel samples were recorded using X-Pert PRO X-Ray Diffractometer (PANalytical) (Malvern Panalytical Ltd, Malvern, United Kingdom) with Ni-filtered Cu Kα radiation (λ = 1.5418 Å) in the 2θ range from 20° to 90°. Throughout XRD diffractograms, the identification of iron and chromium oxides specific peaks will refer to the International Centre for Diffraction Data (ICDD) cards as follows: $Fe_{2}O_{3}/Fe_{3}O_{4}$ (00-001-1053, 00-002-0919, 00-013-0458 and 00-005-0637) and $Cr_{2}O_{3}$ (00-01-1294 and 00-002-1362).

The topographical measurements of the sample surface have been carried out using the confocal laser microscope (LEXT OLS4000 from Olympus, Olympus Corporation, Tokyo, Japan). The measurements were performed for two objective lenses of x20 and x100. The 3D surface roughness parameters $S_{a}$ (average surface roughness) and $S_{q}$ (root mean square roughness) were calculated based on measurements recorded for the objective lens x20, according to the equations

(1) $$S_{a}=\frac{1}{A}\iint_{A}\left|z(x,y)\right|dxdy,$$

(2) $$S_{q}=\sqrt{\frac{1}{A}\iint_{A}z^{2}(x,y)dxdy},$$

where z(x,y) is the function representing the height of the surface relative to the best fitting mean plane described by area A.

Raman spectroscopy has been carried out in the range of 50–1500 cm^−1^ with a laser wavelength of 532 nm, and a laser source power of 10 W (Senterra, Bruker Optik GmbH, Ettlingen, Germany).

The continuous stiffness measurement (CSM) technique was applied for microhardness and nanohardness identification. The nanoindenter (CSEM Platform with nanoindentation modle, CSEM-Instruments, Peseux, Switzerland) with a Berkovic intender was firstly calibrated using the fused silica according to the standard indentation procedure in the range of 0.1 mN to 1000 mN [33]. The load was applied continuously, reaching the maximum load threshold of 500 mN. The indentation test was performed seven times for each sample, providing a constant 0.3 mm offset between adjacent residuals. The distance was established to avoid measurement artefacts resulting from strain effects occurring around the tip of a particular indentation. Moreover, all of the specimens were indented, maintaining a constant acquisition rate of 10.0 Hz, and reaching the indentation depth of about 3000 nm for the AISI 304 and 4000 nm for the AISI 316. The Poisson’s ratio of tested material was assumed to be 0.3 [34].

The Oliver-Pharr method was applied to determine the local hardness H_IT_ and instrumental Young modulus E_IT_ [35,36]. Within this approach, the loading-unloading curve can be expressed by plotting $P/S^{2}$, where P is the indenter load and S is the contact stiffness. Thus, considering both plastic and elastics deformations, the nanoindentation hardness can be defined as follows [37]

(3) $$H=\frac{P_{\max}}{A},$$

where $P_{\max}$ is the maximum load and A is defined as the contact area between the indenter and the specimen evaluated for the maximum indenter depth. The first estimate of projection area A for an ideally sharp indenter with indentation depth $h_{c}$ can be expressed as [37]

(4) $$A=C_{0}h_{c}^{2},$$

where $C_{0}$ for the Berkovic indenter is 24.5 [37].

For the sake of instrumental elastic modulus determination, the reduced modulus $E_{r}$ is used to incorporate the elastic deformation effect between the tested specimen and the indenter. Hence, *E_IT_* can be calculated from $E_{r}$ by the given equation [38]

(5) $$\frac{1}{E_{r}}=\frac{1-\nu_{i}^{2}}{E_{i}}+\frac{1-\nu^{2}}{E_{IT}},$$

where ν represents the Poisson’s ratio of the sample and $\nu_{i}$ and $E_{i}$ are the Poisson’s ratio and elastic modulus of the indenter, respectively. The quantities for the diamond tip material are $E_{i}=1140$ GPa and $\nu_{i}=0.07$ [38].

Consequently, the contact stiffness S can be linked with a reduced elastic modulus, as follows [39]

(6) $$E_{r}=\frac{\sqrt{\pi }}{2\beta }\frac{S}{\sqrt{A}},$$

where β is the indenter geometry-related constant, and for the Berkovic tip β=1.034 [39].

## 3. Results and Discussion

### 3.1. Topography

**Figure 1:**
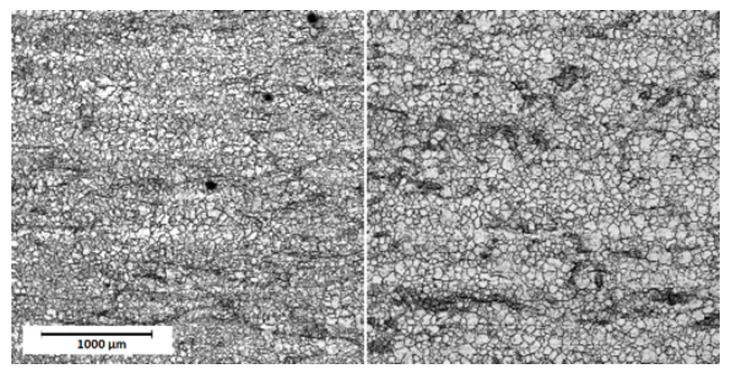
The confocal microscopy images of the untreated AISI 304 (**left**; 304_RAW) and 316 (**right**; 316_RAW) stainless steel plates.

**Figure 2:**
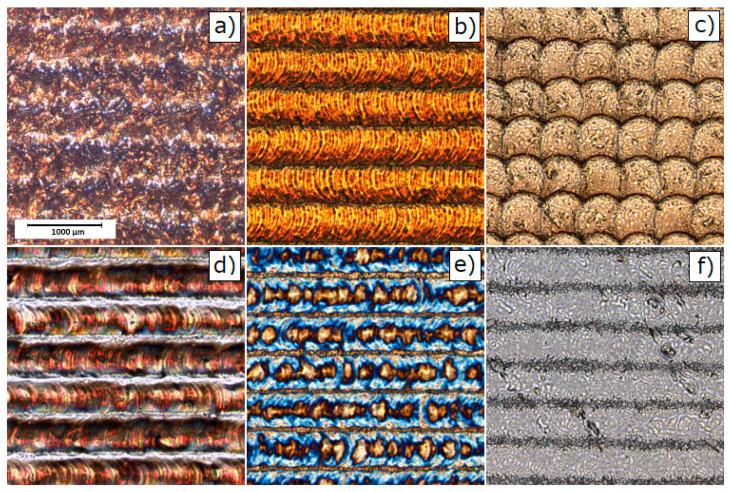
The AISI 304 COM images of surfaces treated with different laser marking parameters (i.e., scanning speed, pulse repetition rate) recorded for: (**a**) 304_20_10; (**b**) 304_20_100; (**c**) 304_20_1000; (**d**) 304_80_10; (**e**) 304_80_100; and (**f**) 304_80_1000 samples (naming of samples: see Table 2).

**Figure 3:**
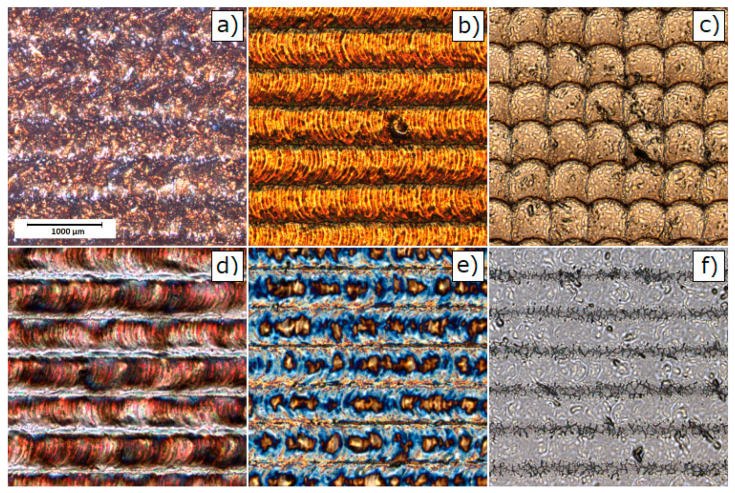
The AISI 316 COM images of surfaces treated with different laser marking parameters (i.e., scanning speed, pulse repetition rate) recorded for: (**a**) 316_20_10; (**b**) 316_20_100; (**c**) 316_20_1000; (**d**) 316_80_10; (**e**) 316_80_100; and (**f**) 316_80_1000 samples (naming of samples see: Table 2).

**Figure 4:**
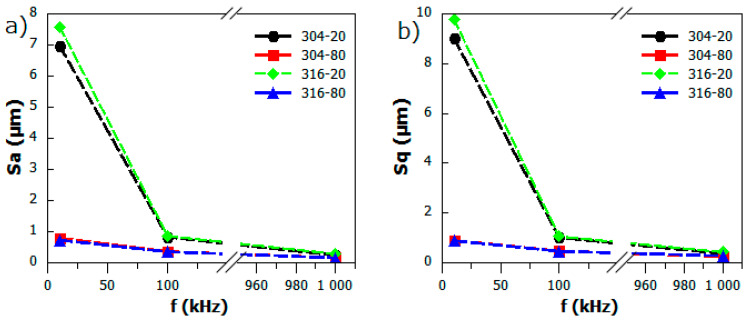
The (**a**) S_a_ and (**b**) S_q_ 3D roughness parameters versus pulse repetition rate (f) for laser-treated AISI 304 and 316 sample series.

Figure 1 presents representative COM images for untreated AISI 304 and 316 stainless steel. The pulsed laser proceeded stainless steel surfaces are shown in COM micrographs on Figure 2 for AISI 304 and Figure 3 for AISI 316. The clear single overlapping traces can be reported for most of presented images. However, for the edge laser treatment parameters—i.e., the scanning velocity v = 20 mm/s and the pulse frequency f = 10 kHz—traces become fuzzy and the sharp intermediate area between the two adjacent grooves vanishes. Moreover, at low frequencies and a high level of scanning speed, each single pulse can be easily distinguished as a particular dot in a marking line. Given the fact that some of the modified samples exhibit a high level of anisotropy, authors decided to evaluate surface roughness using the 3D surface roughness parameters $S_{a}$ and $S_{q}$. Table 3 presents the results of 3D roughness parameters obtained for laser-treated samples and the raw steel. The 3D roughness parameters of untreated (raw) steel are $S_{a}=0.209$ µm; $S_{q}=0.372$ µm for AISI 304 and $S_{a}=0.166$ µm; and $S_{q}=0.325$ µm for AISI 316. As can be seen from Table 3, surface roughness measurements revealed that the overlapping distance between two pulses—obviously related with treatment parameters—directly affects surface smoothness. The overall roughness of laser-treated layers decreases with the rises in the pulsed repetition rate and scanning speed. The collected parameters range from 0.162 µm to 6.977 µm and 0.255 µm to 9.018 µm for the 304 steel and from 0.166 µm to 7.587 µm and 0.325 µm to 9.776 µm for 316 steel considering $S_{a}$ and $S_{q}$, respectively. Figure 4 compares the calculated roughness parameters for the 304 and 316 sample series. It is apparent from Figure 4 that the highest degree of surface roughness were observed for samples treated with a scanning speed v = 20 mm/s and a repetition rate f = 10 kHz, while the lowest were reported for a scanning speed v = 80 mm/s and a repetition rate f = 1000 kHz for both the 304 and 316 sample series. The significant drop in $S_{a}$ and $S_{q}$ parameters are most probably related to the accumulation of energy effect, which was observed earlier and was described in the literature [40,41]. The relatively high level of surface smoothness is achieved by overlapping multiple treatment cycles. The cross-section examination, perpendicular to the marking lines direction, indicates that the regions proceeded with low scanning speed values and low pulses repetition rates, resulting in deeper penetration and higher debris accumulation formed between particular grooves.

### 3.2. Phase Composition

**Figure 5:**
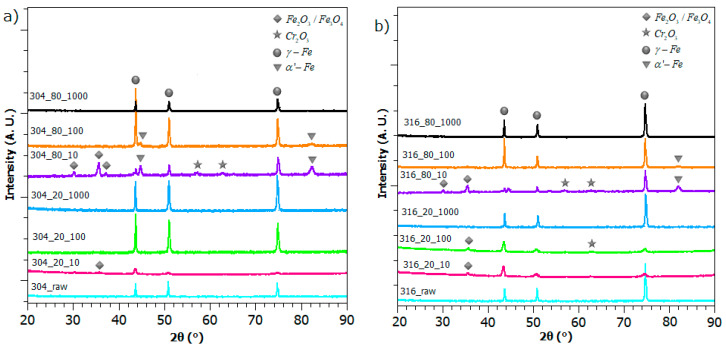
The XRD patterns of laser modified samples and untreated steel: (**a**) AISI 304; and (**b**) AISI 316 stainless steel.

The X-ray diffraction (XRD) results of both stainless steel AISI 304 and 316 are shown in Figure 5a,b respectively. In general, the chemical state of AISI 304 and 316 laser-treated samples remains very similar, as reflected in the particular compounds’ peak position. The XRD spectra for both untreated stainless steel samples revealed the presence of authentic phases (FCC) for three major peaks (2θ = 43.6°; 51°; 74.8°). Moreover, it can be seen that main γ-Fe peaks persist for each irradiated sample within slight changes in its intensity. This appears to be in accordance with results reported by different authors [42,43,44,45]. Alongside austenitic specimens, the α’-Fe peaks (2θ = 44.7°; 82.3°) can be reported for two samples, structured with pulsed frequencies of f = 100 kHz and f = 10 kHz and a scanning speed of v = 80 mm/s. A recent study by Singh et al. reveals that austenite phases can even transform to α’ - martensite under cryogenic deformation [43].

XRD patterns also show a presence of $Fe_{2}O_{3}/Fe_{3}O_{4}$, which corresponds to peaks measured for 2θ = 30.2°; 35.6° and 2θ = 62.7°, analyzing both 304 and 316 stainless steel. These $Fe_{2}O_{3}/Fe_{3}O_{4}$-attributed peaks can be observed for each laser proceeded sample except these, which were modified with a pulsed frequency f = 1000 kHz. As demonstrated by Kucera et al. [42], the intensity of corresponding iron oxide peaks increase with the rise of heat input, and for values above 1.0 J mm^−2^, $Fe_{3}O_{4}$ compounds can be detected. The results of XRD analysis also show specimens with a presence of $Cr_{2}O_{3}$ (2θ = 57.2°; 65.2°).

**Figure 6:**
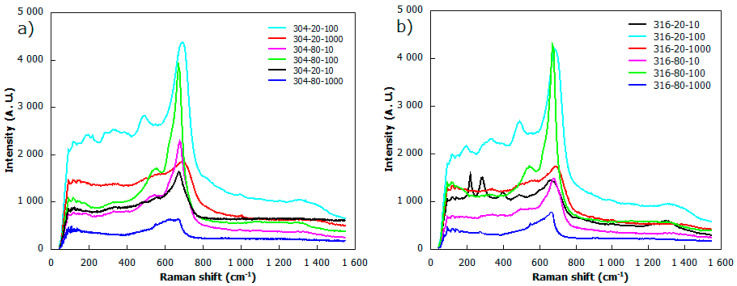
The Raman spectra of laser treated samples for: (**a**) AISI 304; and (**b**) AISI 316 stainless steel.

Figure 6 presents the Raman characterization spectra of the laser-treated specimens. The Raman spectra of treated samples gives the evidence of the most prominent bands at around 470 cm^−1^, 560 cm^−1^ and 670 cm^−1^, which are attributed to $Fe_{3}O_{4}$ and $\gamma -Fe_{2}O_{3}$. These iron oxide specimens were also reported by other authors in [23,24,46]. The peak cantered at around 1350 cm^−1^ can also be associated with $Fe_{3}O_{4}$ formation [46]. Peaks at around 220 cm^−1^, 280 cm^−1^ and 400 cm^−1^ could be attributed to $\alpha -Fe_{2}O_{3}$ phase, and are consistent with those established in the work of Wang et al. [25]. Surprisingly, there is no significant contribution of chromium oxide that was reported in previous studies [47] for the Raman band at 820 cm^-1^. The evidence from the Raman spectra study suggest that laser modified samples mainly consist of chromium and iron oxides in the subsurface zone.

### 3.3. Microhardness Analysis

**Figure 7:**
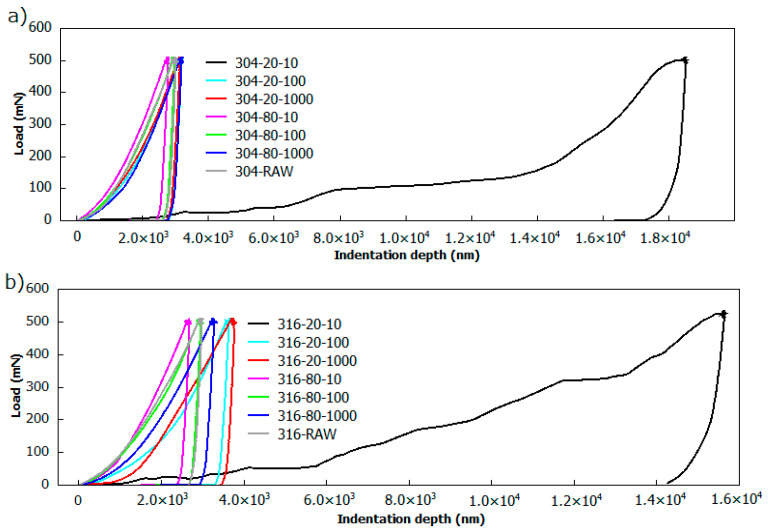
The load-displacement curve plots for: (**a**) AISI 304; and (**b**) AISI 316 stainless steel samples.

The indentation curves of AISI 304 and 316 laser-treated stainless steel are presented in Figure 7. Considering the maximum force threshold applied for examination purposes, it was found that adjusting the maximum load to 500 mN delivers the most reproducible results, and enables the tracking of hardness changes in regions of small depths, as well as substrate-affected ones. Under the continuous material examination, the resulting load versus indentation depth F(h) characteristics were used to determine the instrumental hardness H_IT_ and instrumental elastics modulus E_IT_. It can be noticed that the penetration depth increases slightly with pulsed duration, and does not exceed 3000 nm for every irradiated sample of the AISI 304 series, and 4000 nm for the AISI 316 series of samples. Unlike the other, the indentation profiles of steels proceeded with lowest pulse repetition rate, and the scanning speed exhibits the indentation depth, reaching 16,000 nm and 18,000 nm for AISI 304 and 316 steel, respectively. This yields to be even 6–8 times higher than for the other of the samples. In particular, the analysis of these samples was problematic. The rippling nature of the 304_20_10 and 316_20_10 load curves demonstrates a high degree of inhomogeneity, manifesting indenter-oscillatory feature discontinuity [48]. These discontinuities point out the soft and fragile nature of tested material. This huge difference in penetration depth is attributed to a relatively high level of energy delivered through each pulse, and the high pulse concentration resulting from a low scanning speed.

**Figure 8:**
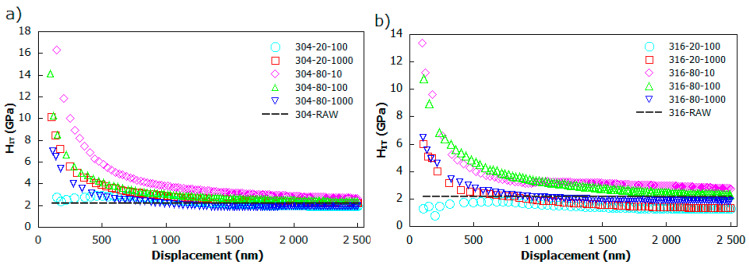
The scatter of nanoindentation hardness H_IT_ versus indentation depth of: (**a**) AISI 304; and (**b**) AISI 316 laser-treated stainless steel samples.

Figure 8 presents the indentation hardness (H_IT_) profile as a function of indentation depth for both AISI 304 (Figure 8a) and AISI 316 (Figure 8b) stainless steel. The experimental results of indentation depths of less than 80 nm were neglected for the sake of surface contamination artefacts and uncertainty resulting from the indenter tip geometry [49]. The trend for both samples series is similar—with an increase in irradiation depth, a rapid decrease of hardness can be observed. The effect of laser modification hardness enhancement can clearly be observed, especially as the indentation depth varies between 100–1000 nm for all exanimated samples. Table 4 provides the maximum values of H_IT_ and average ones, which were established in the region of a substrate-effect with a penetration depth ranging from 1000–2500 nm. These results yield maximum hardness H_IT_ = 16.32 GPa for the 304_80_10 sample at 146 nm depth. Consequently, the maximum value of hardness H_IT_ = 13.36 GPa for the indentation depth of 100 nm was attained to the 316_80_10 sample. Exceeding the average 1 μm of indentation, depth curves become flat and persist at the same level, reaching 2.5 μm, with a corresponding maximum average hardness of 3.01 GPa for both the AISI 304 and AISI 316 series of samples, respectively. This value is slightly higher than the bulk hardness of untreated stainless steel (around 2.2 GPa), and can be treated as the maximum average hardness influenced highly by untreated substrate. The measured base hardness for AISI 304 and AISI 316 stainless seems to be in accordance with the study performed by Chang Ye et al. [50], and with those conducted by Lang et al. [51]. Based on indentation hardness profiles, the evaluated thickness of laser proceeded layers reached a maximum of 1000 nm, depending on the modification process parameters. Overall, analyzing obtained scatters, one can see that the hardening phenomenon provides direct evidence of hardness enhancement for the highest considered scanning speed v = 80 mm/s and the lowest value of pulse repetition frequency f = 10 kHz. Interestingly, increasing the pulse frequency does not lead to improvements in hardness.

**Figure 9:**
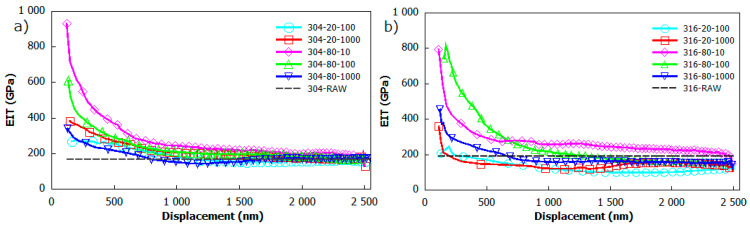
The dependence of elastics modulus (E_IT_) corresponding to indentation depth of: (**a**) AISI 304; and (**b**) AISI 316 stainless steel.

Figure 9 demonstrates changes in reduced elastic modulus versus the indentation depth for (a) AISI 304 and (b) 316 stainless steel. Compared to hardness curves, a similar drop in reduced modulus for the indentation depth in a range of 100–1000 nm can be observed. As all curves follow the same trend, some of these drop sharply, exceeding the region of subtract effect enhancement at around 500 nm when others fall in a smooth manner. In the region at around 800 nm below the surface, the reduced modulus values saturated at around some constant level, and finally, curves indicated by the subtract effect become flat. Generally, the highest hardness and reduced modulus of irradiated samples occurs in the region of small depths. The maximum and average values of reduced modulus (E_IT_) were summarized in Table 4. The maximum value of reduced modulus E_IT_ was observed for the 304_20_10 sample, with an indentation depth of 118 nm and around 931 GPa. Furthermore, the maximum reduced modulus of 823 GPa was obtained for the 316_20_100 sample, with an indentation depth of 172 nm. The average values of reduced modulus determined for the irradiation depth ranging from 1000–2500 nm are summarized in Table 2. These results are significantly influenced with respect to the substrate effect, and are in accordance with those reported in [51,52,53]. These results revealed the heterogeneity of elastics properties, which are strongly influenced by hard oxide formation caused by laser irradiation.

## 4. Conclusions

The present study was designed to determine micromechanical and microstructural properties of specimens formed by Nd:YAG (1064 nm) pulsed laser irradiation on stainless steel substrates. Overall, the relationship between laser irradiation induced changes in morphology, microstructure and micromechanical properties as matters of process parameters estimation were discussed. The produced samples were treated, varying the pulse repetition rate and scanning speed of the pulsed laser source. The investigation of nanoindentional hardness and elastic modulus demonstrates an evident relationship between laser penetration depth and determined H_IT_ and E_IT_ values. The rapid decrease in hardness and elastic modulus, with a rise in irradiation depth, can also be observed. The hardness improvement in a small depth region (100–200 nm) can be attributed to iron and chromium oxide formation, which is apparent from X-ray diffraction (XRD) and Raman spectroscopy analysis. The most obvious finding to emerge from this study is that the scanning speed and pulse repetition rate directly affect the surface roughness and micromechanical properties of considered samples. The increase in pulse repetition frequency leads to a lower level of debris accumulation, resulting in a smoother surface.

## Figures and Tables

**Table 1:** Chemical composition of AISI 304 and 316 stainless steel [32].

Elements (wt. %)	C	Mn	Si	P	S	Cr	Ni	N	Mo
AISI 304	0.08	2.0	0.75	0.045	0.03	20.0	10.5	0.1	–
AISI 316	0.08	2.0	1.0	0.045	0.03	18.0	14.0	0.1	3.0

**Table 2:** Laser marking process parameters (v—scanning speed, f—pulse repetition rate).

Sample	v (mm/s)	f (kHz)
304_20_10	20	10
304_20_100	20	100
304_20_1000	20	1000
304_80_10	80	10
304_80_100	80	100
304_80_1000	80	1000
316_20_10	20	10
316_20_100	20	100
316_20_1000	20	1000
316_80_10	80	10
316_80_100	80	100
316_80_1000	80	1000

The m_k_l sample naming convention was adopted for the purpose of this study, and refers to: m—steel grade; k—scanning speed; and l—pulse repetition rate.

**Table 3:** The 3D surface roughness parameters S_a_ and S_q_. The 304_RAW and 316_RAW samples represent the unmodified plates.

Sample	S_a_ (µm)	S_q_(µm)
304_20_10	6.977	9.018
304_20_100	0.825	1.014
304_20_1000	0.241	0.368
304_80_10	0.792	0.900
304_80_100	0.357	0.471
304_80_1000	0.162	0.255
304_RAW	0.209	0.372
316_20_10	7.587	9.776
316_20_100	0.858	1.056
316_20_1000	0.284	0.424
316_80_10	0.727	0.884
316_80_100	0.353	0.463
316_80_1000	0.160	0.278
316_RAW	0.166	0.325

**Table 4:** The maximum nanoindentaion hardness H_IT_, maximum elastic modulus E_IT_ and average H_IT_ and E_IT_ obtained for the indentation depth range of 1000–2500 nm.

Sample	HIT_MAX_ (GPa)	EIT_MAX_ (GPa)	HIT_AVG_ (GPa)	EIT_AVG_ (GPa)
304_20_100	2.95 ± 0.6	296 ± 32	2.11 ± 0.2	159 ± 23
304_20_1000	10.15 ± 1.2	384 ± 41	2.48 ± 0.2	183 ± 31
304_80_10	16.32 ± 2.4	931 ± 111	3.01 ± 0.3	209 ± 40
304_80_100	14.13 ± 2.1	610 ± 74	2.54 ± 0.2	187 ± 33
304_80_1000	7.05 ± 1.0	340 ± 48	1.96 ± 0.2	165 ± 27
304_RAW	–	–	2.63 ± 0.2	167 ± 9
316_20_100	1.84 ± 0.2	287 ± 32	1.35 ± 0.1	106 ± 15
316_20_1000	6.02 ± 0.9	359 ± 43	1.51 ± 0.1	144 ± 28
316_80_10	13.36 ± 1.9	794 ± 84	3.01 ± 0.2	235 ± 47
316_80_100	10.72 ± 2.1	823 ± 101	2.59 ± 0.3	165 ± 27
316_80_1000	6.47 ± 1.3	454 ± 69	1.98 ± 0.1	158 ± 27
316_RAW	–	–	2.21 ± 0.2	258 ± 28
